# Transforming tabular data into images via enhanced spatial relationships for CNN processing

**DOI:** 10.1038/s41598-025-01568-0

**Published:** 2025-05-16

**Authors:** Hameedah A. Alenizy, Jawad Berri

**Affiliations:** 1 Information Systems Department, College of Computer and Information Sciences, King Saud University, Riyadh, 11451 Saudi Arabia; 2 Department of Computer Science programs, Applied College, Princess Nourah bint Abdulrahman University, Riyadh, 13414 Saudi Arabia

**Keywords:** NCTD, Tabular data, Image generation, Convolutional neural networks, Image classification, Applied mathematics, Computational science, Computer science, Information technology

## Abstract

Convolutional neural networks (CNNs), renowned for their efficiency in image analysis, have revolutionized pattern and structure recognition in visual data. Despite their success in image-based applications, CNNs face challenges when applied to tabular data due to the lack of inherent spatial relationships among features. This weakness can be overcome if the original tabular data is expanded to create an enhanced image that exhibits pseudo-spatial relationships. This paper introduces an original approach that transforms tabular data into a format suitable for CNN processing. The Novel Algorithm for Convolving Tabular Data (NCTD) applies mathematical transformations including rotation translation and reflection, to simulate spatial relationships within the data, thereby constructing a data structure analogous to a 2D synthetic image. This transformation enables CNNs to process tabular data efficiently by leveraging automated feature extraction and enhanced pattern recognition. The NCTD algorithm was extensively evaluated and compared with traditional machine learning algorithms and existing methods on ten benchmark datasets. The results showed that NCTD consistently surpassed the majority of competing algorithms in nine out of ten datasets, indicating its potential as a robust tool for extending CNN applicability beyond conventional image-based domains, particularly in complex classification and prediction.

**Supplementary Information:**

The online version contains supplementary material available at 10.1038/s41598-025-01568-0.

## Introduction

Convolutional neural networks (CNNs) are a class of deep neural networks that use multiple layers to capture different aspects of images, making them highly effective in recognizing patterns and structures, particularly in image processing tasks^1,2^. This has led to widespread adoption in applications such as image classification, object detection, and video analysis^3^. The success of CNNs in pattern recognition lies in their ability to automate feature extraction, a task that traditionally required substantial human effort in conventional machine learning workflows^4^. CNNs are particularly powerful for tasks involving visual perception, and their performance can often be enhanced with larger, high-quality datasets and sufficient computational power.

The use of CNNs for tabular data is unconventional because CNNs are typically used for spatial data where spatial hierarchies are relevant. Nevertheless, CNNs can be creatively applied to tabular data by capturing patterns and relationships that can be represented spatially. Transforming tabular data into synthetic images offers the potential to improve model performance by adding 2D spatial information, which CNNs process efficiently. For datasets with inherent classification challenges, CNNs alone may not significantly improve performance. However, converting tabular data into synthetic images allows CNNs to leverage their 2D processing capabilities, achieving results comparable to traditional models optimized through grid search and feature selection.

Many proposed techniques^5,7–10,14–23^ (see Related Work Section) aim to generate patterns within synthetic images by processing the tabular data features in various ways. These approaches focus on identifying and leveraging correlations and similarities between features across all samples. To effectively map these relationships, some methods use dimensionality reduction methods to determine the most optimal 2-D coordinates for each feature^5,15,17^. Other studies^8–12^, have used dimensionality reduction methods, which implicitly include data iteration to achieve optimal reduction results; this process sometimes is computationally expensive in terms of both memory and processing time. Furthermore, the effectiveness of these algorithms heavily depends on the relevance of the computed correlations. When features exhibit nonlinear relationships that correlation coefficients fail to capture, these methods may require modifications or enhancements to achieve better performance.

This study presents key contributions to the field of data transformation and deep learning. First, it provides a comparative analysis of different tabular-to-image transformation techniques, assessing their effectiveness across benchmark datasets. Second, it introduces a novel direct approach (NCTD) for converting tabular data into synthetic images, preserving key relationships within the data. Finally, it leverages the power of CNNs to analyze these synthetic images, enabling the application of CNNs’ pattern recognition capabilities to tabular data efficiently as demonstrated in the experiments, where NCTD consistently surpassed the majority of competing algorithms. The proposed approach opens new opportunities for applying deep learning to tasks relying deeply on tabular data such as medical diagnostics and financial forecasting.

The structure of this paper is as follows: Sect. 2 provides a detailed review of related work, discussing existing methods for transforming tabular data and their applications in machine learning. In Sect. 3, we introduce the proposed method, detailing a novel approach for converting tabular data into synthetic images using mathematical transformations. Section 4 presents the experimental setup, including datasets, evaluation metrics, and a comparative analysis of various tabular-to-image transformation techniques. Experiment results are discussed in Sect. 5, where we evaluate the performance of the proposed method and compare it with traditional machine learning models. Finally, Sect. 6 concludes the paper and discusses potential avenues for future research, including the refinement of the method and the exploration of hybrid models.

## Related work

The literature highlights various methods for converting tabular data into synthetic images, utilizing diverse techniques to link traditional tabular data formats with the advanced pattern-recognition capabilities of CNNs, which are inherently adept at processing structured image data. This research paves the way for expanding the application of deep learning techniques to domains that predominantly feature tabular data, leveraging the robustness and precision of these methods. The algorithms developed and implemented in this study can be broadly classified into six distinct categories.

### Data wrangling techniques

Sharma and Kumar^6^ introduced three fundamental data-wrangling techniques to preprocess numerical tabular data from breast cancer studies for conversion into image format: an equidistant bar graph, the normalized distance matrix, and a hybrid method combining both. When processed through VGGNet16, the transformed data exhibited significantly higher classification accuracy than that achieved with traditional artificial neural networks, suggesting considerable scope for further refinement and enhancement of these techniques.

### Dimensionality reduction-based approaches

In these approaches^5,8–12^, methods such as t-distributed stochastic neighbor embedding (t-SNE), kernel principal component analysis (PCA), or Fourier transform are used to reduce high-dimensional tabular data to 2D^11^. Then some approaches apply Convex Hull algorithm to enclose the projected data points^5,10–12^.

t-SNE is a dimensionality reduction technique that computes high and low dimensional similarity matrices to represent data in lower dimensions. In the high-dimensional space, similarities between data points are measured using a Gaussian similarity function where closer points have higher similarity. In the low-dimensional Convex Hull, an algorithm is used to find the smallest convex shape that encloses all the points, helping identify patterns more clearly. It sorts points by coordinates and employs the Graham scan method to construct the hull, ensuring convexity using cross-product checks. Starting from the point with the lowest x-coordinate, the algorithm iteratively adds points while maintaining the convex shape of the polygon using a stack. Each addition is checked to ensure that convexity is preserved, as determined by the cross products. Points that do not contribute to the convex hull are excluded. This process continues until all points are processed, producing the smallest convex polygon that encloses the original set^11^. The points are then plotted on a 2D matrix for further processing in the neural networks.

The DeepInsight algorithm proposed by Sharma et al.^5^ transforms tabular data into an image-like format, enabling the application of CNNs to traditionally non-image data. This allows models to uncover complex patterns and interactions that are difficult to extract using standard techniques^5^. DeepInsight leverages dimensionality reduction and manifold learning to project high-dimensional data onto a 2D grid, where spatial proximity among features reflects their similarity. Each feature is represented as a pixel in a grayscale image, with pixel intensity corresponding to feature values. By representing feature relationships as spatial arrangements in the resulting images, the algorithm preserves the inherent structure of the data. The system offers flexibility by allowing users to manually select parameters such as the dimensionality reduction algorithm and the image size. Additionally, it incorporates various discretization methods to map features into pixels and address potential overlaps, even when the number of features exceeds the available pixels.

TINTO^7^ is a technique that converts tabular data into images using PCA and t-SNE for classification with CNNs. The blurring technique in TINTO draws inspiration from plastic art, where it is used to soften and extend strokes uniformly, thereby enhancing the characteristic pixels while preserving their intensity. The image-rendering process in TINTO involves generating images based on characteristic pixels, both with and without the blurring technique. When applied, the blurring technique expands the area of the characteristic pixels without diminishing their intensity.

In Fotomics^10^, the authors employ a fast Fourier transform to map features onto a 2D Cartesian plane. After preprocessing, each column undergoes a discrete Fourier transformation, converting it into a sequence of complex numbers represented as $\:a+bi$, where $\:a$ and $\:b$ are the real and imaginary parts, respectively. The real part $\:a$ is plotted along the x-axis, while the imaginary part $\:b$ is plotted along the y-axis^10^.

The authors of^14^ proposed FC-Viz, a method that transforms tabular data into color-encoded images for visualization and classification using CNNs. FC-Viz leverages user-oriented data visualization concepts, including pixel-oriented techniques, feature clustering, and feature interactions. It employs agglomerative hierarchical clustering to form feature clusters and selects representative features from these clusters. These representative interactions are calculated and used to arrange clusters on a 2D grid using ant colony optimization. Dimensionality reduction is then applied to reposition features within each cluster, which are subsequently divided into pixels. The pixel values for each data sample correspond to the scaled feature values^14^.

The REFINED method proposed by Bazgir et al.^15^ is governed by a single parameter: the number of iterations. This parameter determines how frequently the hill-climbing algorithm evaluates all features to assess the cost implications of swapping them. By adjusting the number of iterations, users can control the depth of the algorithm’s exploration and the precision of its optimization in minimizing feature exchange costs. REFINED operates in four stages. First, it uses the Euclidean distance matrix of features as a baseline distance measure. Next, the multidimensional scaling algorithm refines the positions of the features within this space, ensuring that each pixel is associated with a single feature. Finally, the hill-climbing algorithm adjusts the layout by minimizing discrepancies and preventing overlaps in the distances between the repositioned features^15^.

### Feature permutation-based approaches

The EDLT method proposed in^16^ reorders features using 0/1 optimization to create an image for each instance. Related features are identified using Pearson’s correlation for continuous data or the chi-squared test for categorical data. These related features are positioned close together in the constructed image, enabling the CNN to learn effective patterns for classification. Experiments on 22 benchmark datasets demonstrated significant performance improvements when applying CNNs to generic datasets, compared to conventional machine learning methods. The primary aim of this research is to enable deep learning for generic data classification, broadening its applicability to diverse datasets.

The IGTD algorithm proposed by Zhu et al.^17^ uses pairwise Euclidean distances to calculates a distance rank matrix R for the features and compares it with a fixed pixel distance rank matrix Q. The algorithm iteratively swaps feature positions to minimize the difference between R and Q. After reordering, each sample’s features are reshaped into an image, where scaled feature values represent pixel intensities. The flexible design of IGTD allows it to adapt to various data types and requirements, including customizable image sizes and shapes^13^.

### Embedding-based approaches

Methods in this category project each data point (instance) from a tabular dataset onto a 2D embedding, resembling an image. These images are then processed by CNNs, enabling the models to learn from the spatially encoded data^18–20^.

BIE^18^ converts the numerical samples into 64-bit floating-point binary representations. These values are rearranged into a matrix, with 0 s depicted as black pixels and 1 s as white pixels. The binary rows collectively form the final image.

SuperTML^19^ offers two modes for generating images: equal and variable font size. In the equal font size mode, all features in the tabular data are displayed with the same font size. In the variable font size mode, feature sizes are adjusted according to their importance. SuperTML also optimizes feature placement to maximize image space utilization without overlapping.

DWTM^20^ dynamically adjusts feature importance for class prediction by calculating weights using methods such as Pearson correlation and chi-square. Features are ranked based on these weights, and their embedding within the image canvas space is adjusted dynamically rather than using a static configuration. More important features are allocated larger areas in the image, with pixel positions assigned accordingly to reflect their significance^20^.

### Three-dimensional architecture approaches

The Table 2 Vox^21^ method explores optimal approaches for converting tabular data into 3D voxel images and identifying the best 3D CNN architecture. In the first stage, features are initially arranged randomly along voxel axes, with similar features clustered to refine the layout. Candidate layouts are evaluated and assigned weights based on their effectiveness, with gradient descent used to identify the configuration that minimizes errors. In the second stage, a differentiable architecture search, a type of neural architecture search, is employed to fine-tune the 3D CNN architecture.

DAFT^22^ enhances CNNs by integrating tabular data with 3D image data, using ResNet as the backbone. The method employs an auxiliary neural network that generates scaling and offset factors to adjust the feature maps. These factors allow DAFT to dynamically scale and shift the feature maps, enriching the CNN’s capabilities.

### End-to-End-based approaches

These approaches embody the concept of end-to-end explainable artificial intelligence models, where the artificial intelligence system operates seamlessly from input to output, while ensuring complete transparency in its decision-making processes at every stage.

The authors in^11^ proposed Vec2image, an explainable artificial intelligence model that was applied to the analysis of type 2 diabetes using human islet single-cell RNA sequencing datasets. The high-dimensional data vectors are converted into pseudo-images. This is achieved by mapping the data into a lower-dimensional space using techniques like t-distributed Stochastic Neighbor Embedding (t-SNE). In these pseudo-images, individual pixels represent specific features (e.g., gene expression levels), and the spatial arrangement reflects the correlations between these features. The pseudo-images are processed using a parallel deep residual neural network (ResNet) architecture for classification tasks. Additionally, an embedded k-nearest neighbor (KNN) algorithm is utilized to prioritize and interpret features, providing insights into the model’s predictions. The model demonstrates satisfactory performance across various classification tasks while offering insights into cell activity changes and disease-related genes.

LM-IGTD builds on IGTD by adding noise to overcome the limitations of converting low-dimensional and mixed-type tabular data to images. It uses Gaussian noise to increase dimensionality and incorporates stochastic noise generation. Grad-CAM is employed for *post hoc* interpretability to understand the CNN’s classification decisions^22^.

HACNet^23^ addresses the need for generating human-interpretable images using easily understandable template images. It creates distinct, interpretable images that closely resemble certain templates. HACNet consists of two main components: a table-to-image attention-based converter and a CNN-based predictor. The predictor takes the generated image, predicts its class label, and computes the cross-entropy error and mean squared error between the generated and template images. The training parameters in HACNet are updated using stochastic gradient descent to minimize the weighted sum of these losses. HACNet’s training proceeds in two phases: First, both the converter and predictor are trained simultaneously. In the second phase, only the predictor is trained using the gradients from the loss function. The prediction accuracy typically improves in this phase due to batch normalization.

## Methods

This study introduces the Novel Algorithm for Convolving Tabular Data (NCTD), a new technique for transforming tabular data into synthetic images, designed to enhance classification performance using CNNs. To evaluate its effectiveness, we conducted two sets of experiments. First, NCTD was compared against conventional machine learning models, including logistic regression (LR)^24^, classification and regression trees (CART)^25^, random forest (RF)^26^, iterative dichotomies 3 (ID3)^27^, XGBoost^28^, and recurrent neural networks (RNNs)^29^. Second, NCTD was benchmarked against six state-of-the-art tabular-to- image transformation techniques: IGTD, DeepInsight, REFINED, TINTO, HACNet, and Fotomics. A detailed description of these methods can be found in the Related Work section.

The evaluation was conducted using ten diverse datasets, ensuring a comprehensive assessment of NCTD across varying numbers of classes, samples, and feature dimensions. Standard classification metrics—accuracy, precision, recall, and F1 score—were used for performance comparison. The following subsections detail the datasets, baseline methods, the proposed NCTD, and the experimental setup.

### The proposed NCTD

Research in the literature has explored the conversion of tabular data into 2D images, making it suitable for processing by CNNs. These studies have primarily focused on identifying correlations between the features in tabular data. This additional feature engineering step is crucial for overcoming the inherent limitations of low-dimensional tabular data. By enriching datasets with additional dimensions through feature engineering, these methods meet the input requirements necessary for CNNs.

The steps involved in converting tabular data into images for the proposed algorithm are described in the next subsections.

#### Normalization

Normalization is a crucial preprocessing step performed prior to data enhancement. Its primary objective is to transform features onto a similar scale, thereby facilitating faster convergence of CNNs during training and enhancing the quality of results.

Let A ∈ ℝ^*m* × *n*^ represent the dataset, where *m* is the number of rows (samples) and *n* is the number of columns (features). For *j = 1 to n*, if *Aj* is a categorical feature, normalization is performed in two steps. At first, label encoding is applied, assigning each unique category a numerical label starting from 0 and incrementing by 1. Next, the encoded values are normalized similarly to numerical features.

For numerical features, datasets often exhibit different ranges. For instance, “age” might span from 0 to 100 years, while “income” could range from 0 to 100,000. Without normalization, features with larger ranges may dominate the training process and overshadow features with smaller ranges. To address this, Min-Max normalization is applied to scale all features to a uniform range of [0, 1]. This ensures comparability across features and balances their influence during training. Min-Max normalization is computed as

1 $$\begin{gathered} Min{\text{ }}\left( {{A_j}} \right){\text{ }}={\text{min }}\left\{ {{{\text{a}}_{{\text{1j}}}},{\text{ }}{{\text{a}}_{{\text{2j}}}}, \ldots .,{\text{ }}{{\text{a}}_{{\text{mj}}}}} \right\}, \hfill \\ Max{\text{ }}\left( {{A_j}} \right){\text{ }}={\text{max }}\left\{ {{{\text{a}}_{{\text{1j}}}},{\text{ }}{{\text{a}}_{{\text{2j}}}}, \ldots .,{\text{ }}{{\text{a}}_{{\text{mj}}}}} \right\}, \hfill \\ {{\text{a}}_{{\text{ij}}}}=~\frac{{{a_{ij}}~ - {\text{Min}}\left( {{A_j}} \right)}}{{M{\text{ax}}~\left( {{A_j}} \right) - ~M{\text{in}}\left( {{A_j}} \right)}}\;\quad \forall {\text{1}} \leqslant i \leqslant m\quad and\quad {\text{1}} \leqslant j \leqslant n. \hfill \\ \end{gathered}$$

#### Convert sample to grayscale image

After normalization, the dataset is converted into grayscale images. Each tabular data row is transformed into a sequence of pixel values scaled to a range of 0–1 by multiplying the normalized values by 255. This scaling facilitates faster convergence during training, as normalized data stabilize gradients and ensure consistent optimization^30^.

For *i = 1 to m* perform

2 $${{\text{A}}_{\text{i}}}={\text{ }}\left\{ {{{\text{a}}_{{\text{i1}}}},{\text{ }}{{\text{a}}_{{\text{i2}}}}, \ldots ,{\text{ }}{{\text{a}}_{{\text{in}}}}} \right\}{\text{ where }}{{\text{a}}_{{\text{ij}}}} \in \left[ {0,{\text{ 1}}} \right]\;\forall \quad {\text{1}} \leqslant j \leqslant n$$

Then, construct the grayscale image G as:

$$~{\text{G }}={\text{ }}\left\{ {{{\text{g}}_{\text{1}}},{\text{ }}{{\text{g}}_{\text{2}}}, \ldots ,{\text{ }}{{\text{g}}_{\text{n}}}} \right\}{\text{ where }}{{\text{g}}_{{\text{j }}=}}{{\text{a}}_{{\text{ij}}}}\forall \quad {\text{1}} \leqslant {\text{j}} \leqslant {\text{n}}$$

where *G* is a grayscale image with pixel intensities between 0 and 1. Each row of the tabular data corresponds to a single grayscale image. The values within a row determine the grayscale intensity of the pixels, and each row forms a linear arrangement of pixels in the image. This arrangement ensures that the order and relationships among the original features are preserved. The resulting grayscale images are structured to support filtering and pooling operations within the CNN.

#### Augmenting an N array to an N × N image

This step augments the initial N-element array (grayscale-transformed tabular data) into an N × N matrix optimized for CNN processing. This transformation preserves original feature values while structuring them into a deep learning-friendly format.

**Fig. 1:**
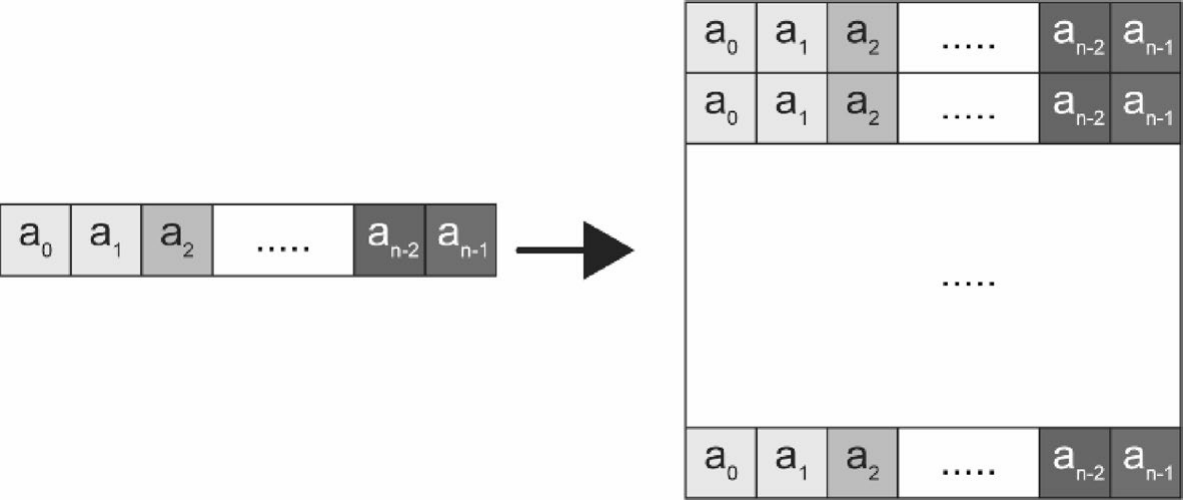
Array Duplication. The N-element array is duplicated across N rows to form an N × N matrix, ensuring that each row maintains the same feature values as the original array.

Three mathematical transformations are applied to construct the *N × N* image. The first transformation involves array duplication, where the N-element array is replicated across N rows, forming an *N × N* matrix in which each row is identical to the original feature array, as shown in Fig. 1.

**Fig. 2:**
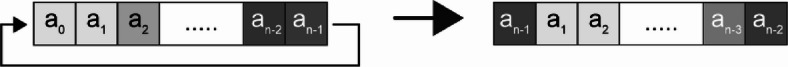
Array Rotation. Each row of the N × N matrix is progressively shifted one position to the left, with the last element repositioned at the beginning. This transformation introduces spatial variation while preserving feature relationships.

The second transformation, array rotation, shifts each row one position to the left, with the last element moving to the first position, as shown in Fig. 2. This process introduces spatial variation while maintaining the relationships between features.

3 $$\begin{gathered} For \:i=1\:to\:m\:do \hfill \\ A=\left[ {\begin{array}{*{20}{c}} {rotate\left( {{A_i},~0} \right)} \\ {rotate\left( {{A_i},~1} \right)} \\ {rotate\left( {{A_i},{\text{~}}2} \right)} \\ . \\ . \\ . \\ {rotate\left( {{A_i},~n - 1} \right)} \end{array}} \right] \hfill \\ \end{gathered}$$

**Fig. 3:**
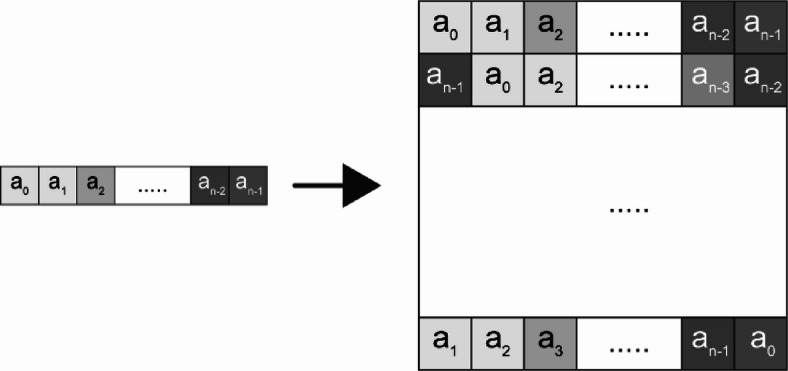
Matrix Formation by Duplication and Rotation. The N-element array is first duplicated across N rows. Then, each row undergoes a leftward shift, increasing progressively from top to bottom. This process generates an N × N grayscale image, effectively capturing multiple perspectives of the same data for CNN processing.

The third transformation combines the previous two steps—duplication and rotation—by forming the N × N synthetic image through replication of the original array across N rows and applying a progressively increasing leftward shift to each row, as shown in Fig. 3.

By combining these transformed versions of the original data row, the final matrix is constructed. By generating multiple perspectives of the same data through sliding and rotation, this approach enhances tabular data representation for CNN-based learning. The effectiveness of these transformations in improving classification performance is evaluated later in the paper.

#### Expanding to a 2 N × 2 N image

**Fig. 4:**
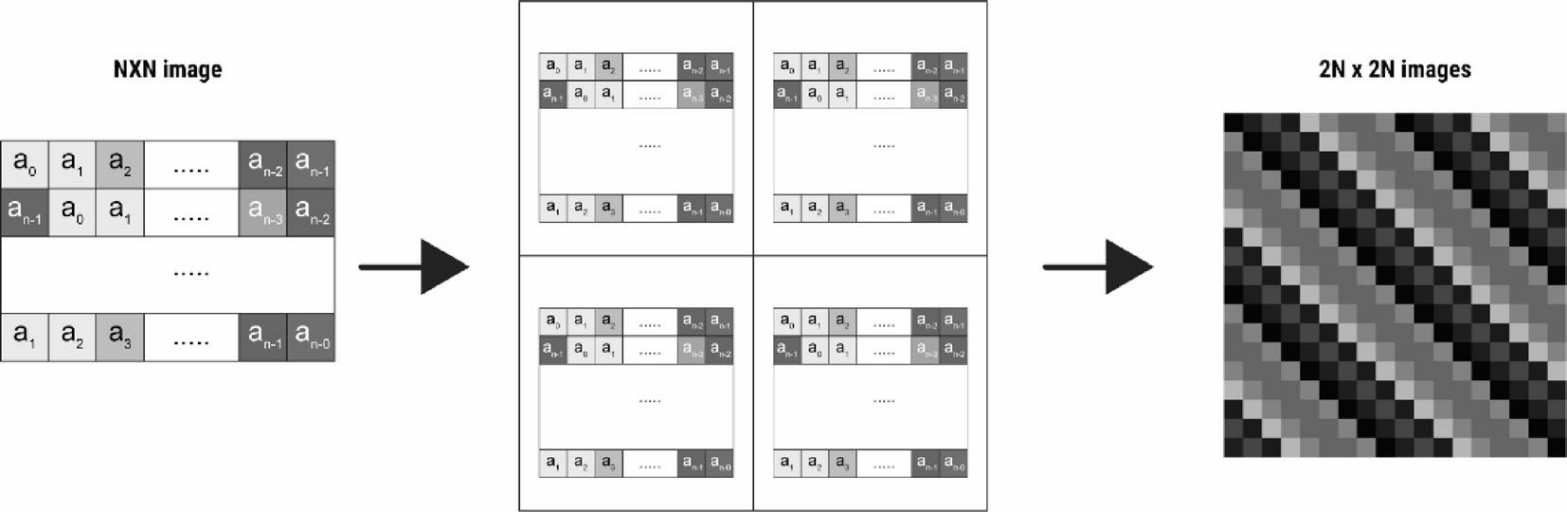
Expanding the N × N image to 2 N x 2 N image.

To enhance the representational capacity of the generated images, the N × N image was positioned in all four quadrants (top-left, top-right, bottom-left, and bottom-right) of a larger 2 *N* × 2 N image. This transformation was applied to each row, resulting in a 2 *N* × 2 N image representation for each data instance. The increased image size provides additional spatial structure, allowing CNN to learn more complex patterns and hierarchical relationships within the data, as shown in Fig. 4.

4 $${{\text{C}}_{\text{i}}}=\left[ {\begin{array}{*{20}{c}} {A~~A} \\ {A~~A} \end{array}} \right]$$

**Fig. 5:**
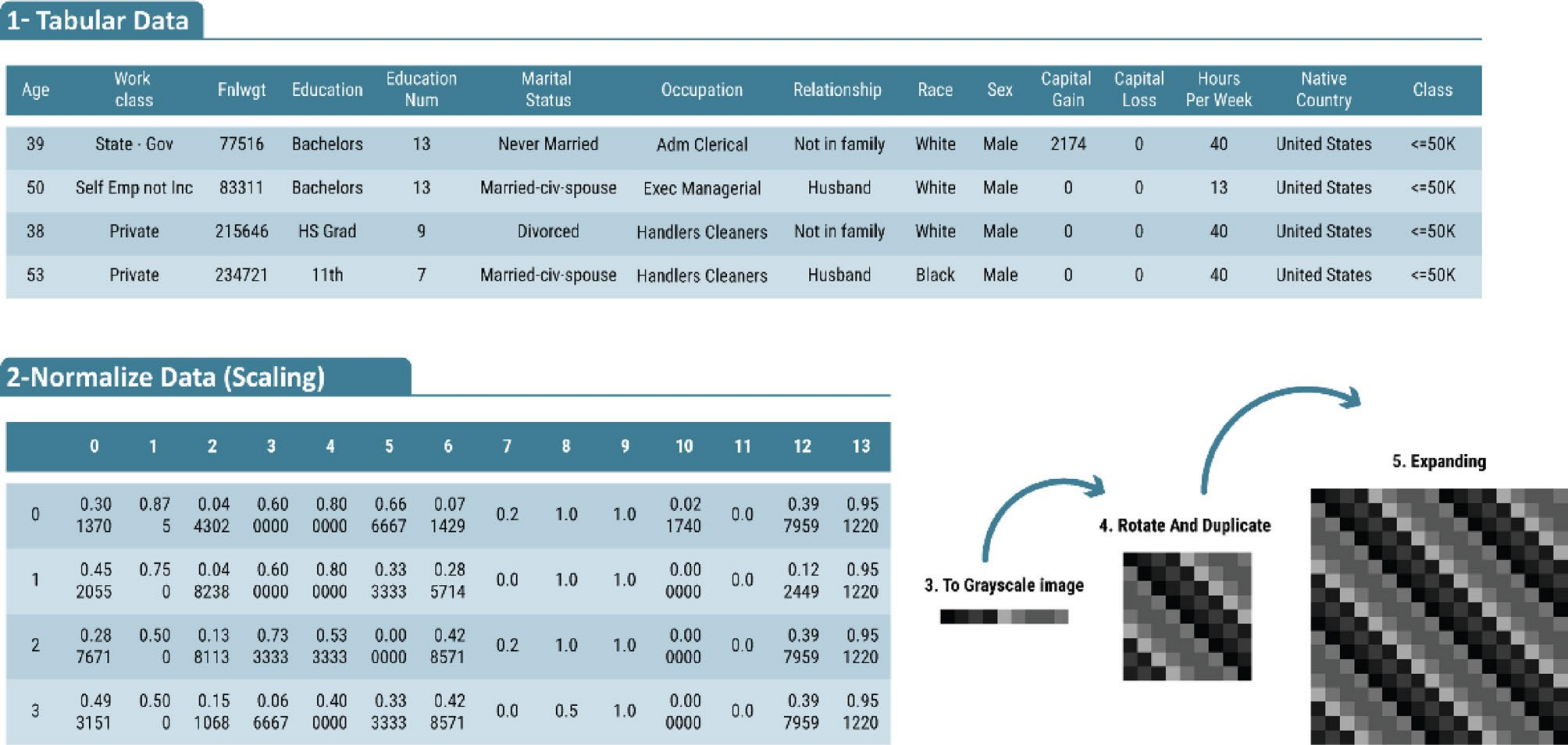
Example illustrating the concept and process of the NCTD (Tabular-to-Image Conversion) algorithm, showing the expansion of a tabular data row into a 2 *N* × 2 N image format.

These steps convert tabular data into a structured grid-like format, which is particularly well-suited for CNNs. When compared with conventional machine learning practices for dealing with tabular data, the new model demonstrates promising accuracy improvements in classification tasks. Figure 5 illustrates an example of the NCTD algorithm, showcasing the transformation process.

### Experimental setup

#### Datasets overview

We evaluated the performance of different algorithms using ten datasets, each presenting a unique prediction task and offering valuable insights into healthcare, education, and socioeconomic analysis. These datasets vary in terms of the number of classes, features, and samples, thus providing a comprehensive testbed for our analysis.

The Wisconsin Breast Cancer Database^31^ aims to predict whether a tumor is benign or malignant based on features derived from biopsy images. In the field of education, the student dropout and academic success^31^ dataset focuses on predicting students’ academic outcomes, such as dropout, enrollment continuation, or successful graduation. In the realm of socioeconomic analysis, the Adult^31^ dataset attempts to predict whether an adult’s yearly income exceeds $50,000, based on demographic and socioeconomic attributes.

In the medical domain, the Heart Disease^32^ dataset aims to predict the presence or absence of heart disease using a combination of medical and lifestyle features. Similarly, the Thyroid Disease^31^ dataset seeks to predict the type or severity of thyroid disorders, and is categorized into distinct classes. The Dengue/Chikungunya^33^ dataset, focuses on infectious diseases and aims to predict the occurrence or prevalence of diseases such as Dengue and Chikungunya based on available features.

The ISOLET^34^ dataset is a collection of speech recordings featuring the spoken alphabet from 150 individuals. Each person pronounced each letter twice, resulting in 52 samples per person for training. Each recording is characterized by 617 features, making it suitable for audio-based classification tasks.

MADELON^35^ and Ringnorm-DELVE^36^ datasets are synthetic. MADELON is a complex dataset with 500 continuous input variables and exhibits nonlinear characteristics. Ringnorm-DELVE contains 7,400 samples, each with 20 features, generated from two distinct multivariate normal distributions. Finally, the RELATHE^37^ dataset is composed of binary text from newsgroup documents with 1,432 samples, each consisting of 4,500 features. A summary of these datasets is provided in Table 1.

**Table 1:** Summary of the 10 datasets used in the experiment, including key characteristics such as the number of classes, samples, and features.

ID#	Dataset Name	# of Classes	# of samples	# of features
DS01	Wisconsin Breast Cancer Database^31^	2	699	9
DS02	Student’s dropout and academic success^31^	3	4,424	36
DS03	Adult^31^	2	32,561	14
DS04	Heart disease^32^	2	918	15
DS05	Thyroid disease^31^	6	9,172	28
DS06	Dengue/chikungunya^33^	3	17,172	26
DS07	Ringnorm-DELVE^36^	2	7,400	20
DS08	ISOLET^34^	26	7,797	617
DS09	MADELON^35^	2	2,600	500
DS10	RELATHE^37^	2	1,427	4322

#### Datasets preprocessing

The datasets used in this study were split into training and validation sets using an 80:20 ratio. This split is a widely adopted practice in machine learning, serving as a baseline for model evaluation rather than a method for mitigating overfitting^38^. Specifically, 80% of the data was used for training the model, while the remaining 20% was reserved for validation. During training, the model was optimized using the training set, and hyperparameters were tuned to minimize validation error. It is important to note that the validation set was not involved in training or model-fitting processes. Classification accuracy was then evaluated on the validation set, providing an unbiased assessment of the model’s performance. Classification accuracy refers to the proportion of correctly classified samples within the validation set. The best performance across multiple epochs was selected to represent the model’s evaluation metrics.

Due to computational resource constraints, PCA was employed to reduce the dimensionality of datasets with a high number of features. This transformation was consistently applied across all methods in the experiment, including both traditional machine learning models and tabular-to-image transformation techniques. Specifically, PCA was used for DS08 (ISOLET), DS09 (MADELON), and DS10 (RELATHE), reducing dimensionality to 200, 450, and 800 principal components, respectively. These component selections retained at least 95% of the total variance^39^ for most datasets, ensuring minimal information loss while significantly improving computational efficiency.

#### Evaluation metrics

To assess the performance of NCTD and compare it with other techniques, we employed several evaluation metrics commonly used in machine learning classification tasks. Accuracy, precision, recall, and F1 score were the primary metrics considered in this study. These measures provide insights into various aspects of model performance, particularly in managing class imbalances and evaluating classification trade-offs.

### CNN architecture and training parameters

**Fig. 6:**
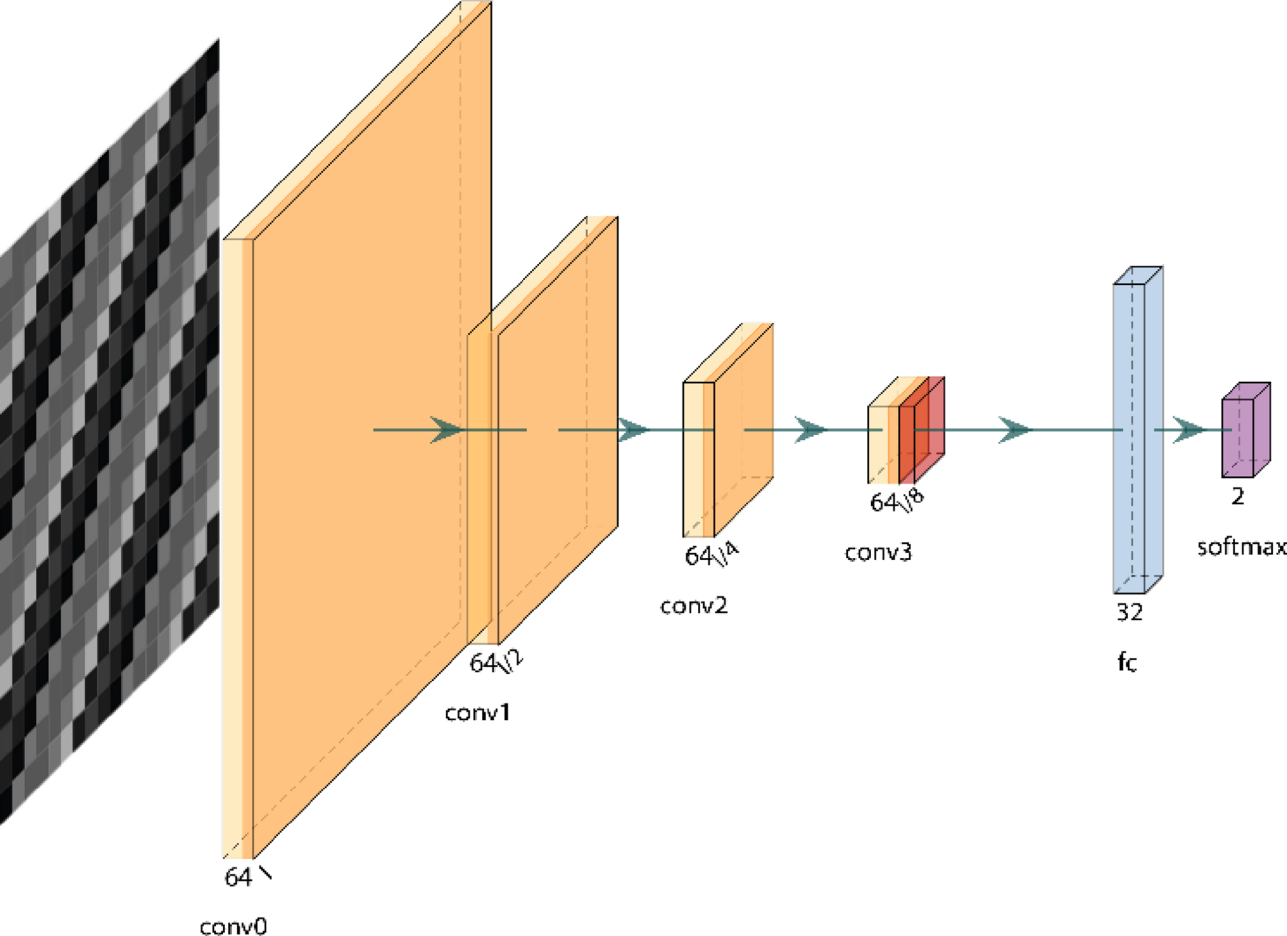
CNN model architecture summary.

A CNN processes an input image, represented as an m × n matrix, by performing feature extraction and classification through various hidden layers, including convolutional, ReLU, and max-pooling layers^39^. Notably, CNNs do not require explicit feature extraction techniques because they automatically derive features directly from raw input. Additionally, CNNs excel at capturing higher-order statistics and nonlinear correlations within images. Convolutional neurons process data within their receptive fields or restricted subareas, reducing the need for a large number of neurons for large input sizes. This allows the network to be deeper and more efficient, with fewer parameters^5^. The same CNN architecture was used for all transformation methods to ensure a consistent basis for comparison across different input representations. Figure 6 illustrates the general CNN architecture used in the experiment.

Convolutional layers are responsible for processing the spatial data in images and detecting features such as edges and textures using filters. The first convolutional layer (conv0) accepts the input and generates feature maps of size 15 × 15 using 64 filters. The subsequent layer (conv1) reduces the spatial dimensions to 7 × 7 while maintaining 64 filters. The next layer (conv2) further reduces the spatial dimensions to 3 × 3, followed by conv3, which reduces the spatial dimensions to 1 × 1. Batch normalization is applied after each convolutional layer to normalize activations, followed by the application of an activation function.

The global pooling layer converts the spatial maps into a one-dimensional vector of size 64 by averaging over the spatial dimensions, yielding a compact representation of the extracted features. The dense layers are fully connected, and all the features detected earlier in the network are used to make the final prediction. The hidden layer is denoted as *r1*, while the final dense layer, *sm*, consists of two units and is used for binary classification tasks.

#### Training parameters for CNNs

Each dataset was trained using a consistent set of parameters, as summarized in Table 2. The datasets encompass various classification tasks with different numbers of classes and features, which were transformed into images of varying sizes for CNN processing. The training parameters included a learning rate of 0.0008, batch size of 64, the Adam optimizer^40^, and categorical cross-entropy^41^ as the loss function.

**Table 2:** Training parameters for the CNN model.

Learning Rate	Batch Size	Optimizer	Loss Function	Epochs
0.0008	64	Adam	Categorical Cross-Entropy	30

### Synthetic images

Several methods exist to convert tabular data into 2D images for CNN analysis. Table 3 shows an illustrative example of how various tabular-to-image conversion methods represent the same data instance from dataset DS01.

**Table 3:**
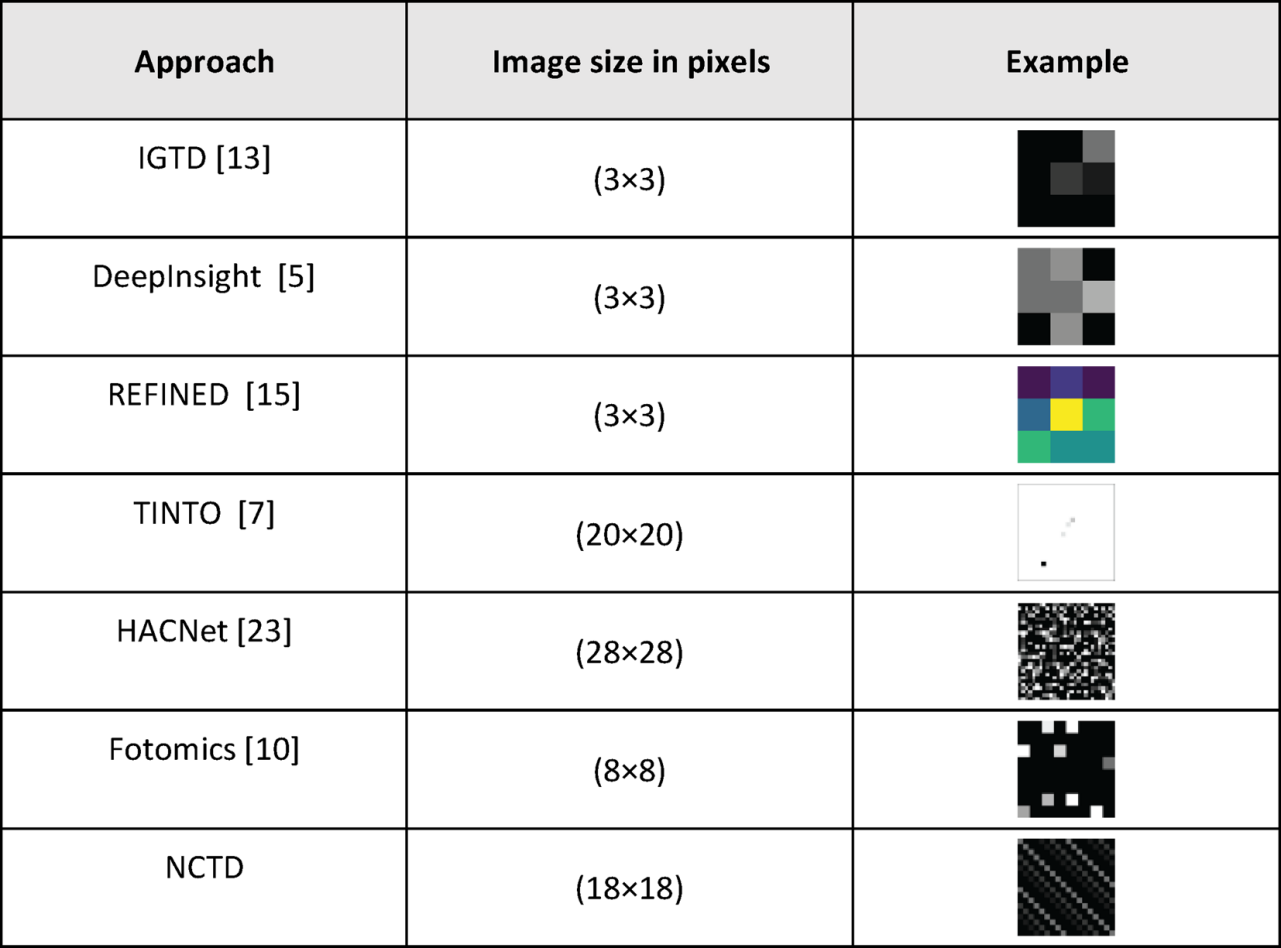
Example illustration of how various tabular-to-image conversion methods represent the same data instance from dataset DS01.

The IGTD algorithm uses pairwise Euclidean distances to iteratively reorder feature positions, minimizing the difference between feature distance rankings. This creates square images where feature values become pixel intensities, with similar features grouped together. Dimensionality reduction-based methods like DeepInsight, TINTO, Fotomics, and REFINED preserve local feature relationships and map them to a 2D space.

DeepInsight and TINTO use nonlinear techniques such as t-SNE and Kernel PCA. DeepInsight employs a convex hull algorithm, while TINTO uses the center of mass of features, applying scaling and blurring to the image. Fotomics utilizes Fast Fourier Transform (FFT) to map features onto a 2D Cartesian plane by converting them into complex numbers. REFINED applies multidimensional scaling and Bayesian optimization to place features as pixels in a 2D map, using a hill-climbing algorithm to reduce overlap.

HACNet focuses on generating interpretable images by using template images. It consists of a table-to-image converter and a CNN predictor. The model generates images that resemble templates, with a two-phase training process aimed at optimizing prediction accuracy.

IGTD typically produces the smallest images across all datasets, while the proposed method often generates the largest images. DeepInsight and Fotomics produce larger, more detailed images compared to IGTD and REFINED. HACNet and TINTO maintain consistent image sizes. While the newly proposed method generates larger images, it may offer more information-rich representations, making them suitable for more complex models.

## Results

Two evaluations were conducted to assess the performance of NCTD. In the first evaluation, NCTD was compared against six conventional machine learning models: LR, CART, RF, ID3, XGBoost, and RNN. These models are widely used for classification tasks and are well-suited for handling large datasets. LR^25^ is a straightforward linear model suitable for binary classification tasks, while CART^26^ uses a decision tree approach for both classification and regression. RF is an ensemble method that employs multiple decision trees to enhance accuracy and mitigate overfitting. ID3 creates decision trees based on entropy and information gain. XGBoost is a highly efficient and scalable implementation of gradient boosting that uses decision trees. RNN is designed for sequence data and temporal dynamics.

In the second evaluation, NCTD was benchmarked against six state-of-the-art tabular-to-image conversion techniques designed for CNN-based classification. These methods—IGTD, DeepInsight, REFINED, TINTO, HACNet, and Fotomics—were discussed earlier. All these methods were implemented and evaluated using the ten datasets listed in Table 3.

### Performance comparison of NCTD with tabular-to-image transformation techniques

In the first evaluation, we compared NCTD against six state-of-the-art tabular-to-image conversion methods: IGTD, DeepInsight, REFINED, TINTO, HACNet, and Fotomics. These methods are specifically designed to transform tabular data into image representations suitable for CNN-based classification. The evaluation was conducted using the ten datasets presented in Table 3.

Table 4 and Appendix A summarize the accuracy, precision, recall, and F1 scores of each method, providing a comprehensive assessment of their effectiveness across various datasets. The objective was to identify the most effective method for each dataset and analyze the strengths and limitations of the different approaches.

**Table 4:** Accuracy comparison of tabular-to-image transformation methods across various datasets.

Dataset	IGTD	DeepInsight	REFINED	TINTO	HACNet	Fotomics	NCTD
**DS01**	95.71%	94.28%	95.00%	90.71%	96.42%	96.42%	**99.28%**
**DS02**	75.48%	62.25%	74.91%	74.01%	75.36%	74.01%	**78.64%**
**DS03**	84.13%	84.66%	85.82%	83.50%	85.50%	85.18%	**86.18%**
**DS04**	83.15%	84.78%	85.86%	83.69%	89.13%	56.52%	**90.22%**
**DS05**	63.21%	64.31%	66.48%	65.55%	70.24%	70.19%	**71.77%**
**DS06**	48.52%	43.58%	61.25%	58.57%	61.89%	57.35%	**62.71%**
**DS07**	95.60%	68.91%	91.28%	94.66%	97.09%	49.52%	**97.70%**
**DS08**	42.69%	87.43%	72.37%	19.93%	**93.26%**	50.38%	70.64%
**DS09**	54.61%	**59.42%**	51.73%	58.07%	57.69%	51.73%	55.57%
**DS10**	61.53%	60.83%	66.08%	60.48%	77.27%	54.54%	**79.37%**

The analysis reveals that NCTD is the most dominant and consistent performer, achieving the highest accuracy in 8 out of the 10 datasets and maintaining the highest average accuracy overall. It is closely followed by HACNet and REFINED, though neither matches NCTD’s breadth of superiority. Notably, NCTD also demonstrates remarkable consistency, with high scores across nearly all datasets. In contrast, Fotomics shows significant performance variance, excelling in DS01 (96.42%) but dropping sharply in DS07 (49.52%) and DS08 (50.38%), indicating potential instability or sensitivity to dataset characteristics.

DS08, in particular, stands out due to its 26-class complexity, resulting in extreme performance differences—TINTO scores only 19.93%, while HACNet achieves 93.26%—highlighting the significant impact of dataset-specific suitability on results. Meanwhile, DS09 and DS10 lack a clear top method, as all approaches yield moderate accuracies. TINTO’s poor performance on DS08 further suggests it may be ill-suited for datasets with high class diversity. On the other hand, REFINED, though rarely the top performer, maintains a stable and reliable level of performance across the board, reflecting its potential value in balanced or general-use scenarios.

### Performance comparison of NCTD with conventional machine learning models

In the second evaluation, the performance comparison across the datasets highlights the strengths of each model, with a particular focus on the superior performance of the proposed NCTD approach. Table 5 and Appendix A present the accuracy, precision, recall, and F1 scores of six conventional machine learning algorithms—LR, CART, RF, ID3, XGBoost, and RNN—alongside NCTD. The datasets used for evaluation vary in the number of classes, samples, and features, ensuring a comprehensive assessment of each algorithm’s effectiveness under diverse conditions.

**Table 5:** Accuracy comparison of conventional machine learning models across multiple datasets.

Dataset	LR	CART	Random Forest	ID3	XGBoost	RNN	NCTD
**DS01**	96.42%	95.00%	95.00%	92.14%	95.00%	98.57%	**99.28%**
**DS02**	74.80%	67.68%	76.27%	71.18%	76.94%	74.57%	**78.64%**
**DS03**	82.38%	80.99%	85.67%	85.42%	86.03%	84.00%	**86.18%**
**DS04**	83.69%	74.45%	84.78%	79.34%	84.78%	87.41%	**90.22%**
**DS05**	67.79%	59.56%	69.70%	66.37%	69.75%	68.22%	**71.77%**
**DS06**	59.70%	50.18%	59.62%	60.96%	61.95%	59.88%	**62.71%**
**DS07**	75.20%	86.01%	95.00%	85.06%	94.72%	96.28%	**97.70%**
**DS08**	**96.41%**	80.76%	94.17%	81.85%	94.87%	3.78%	70.64%
**DS09**	60.19%	77.69%	69.62%	75.00%	**79.80%**	52.30%	55.57%
**DS10**	**90.55%**	85.66%	87.41%	81.81%	88.11%	53.49%	79.37%

In comparison with classical machine learning methods and RNN, NCTD continues to outperform all other approaches, achieving the highest accuracy on 7 out of 10 datasets. Its dominance is especially evident in datasets like DS01, DS02, DS04, DS05, DS06, DS07, and DS10, reinforcing its status as the most consistently high-performing method. While XGBoost and Random Forest often perform competitively—occasionally matching or closely trailing NCTD—their performances are less consistent across datasets. RNN shows notable strength on DS07, where it nearly rivals NCTD, and outperforms all others on DS04, but performs poorly on DS08 and DS10. DS08 is particularly concerning, with a score of only 3.78%, suggesting severe overfitting or incompatibility with that dataset’s structure.

Logistic Regression (LR) demonstrates reliable, moderate performance, ranking competitively on DS01 and DS08, but not surpassing modern methods overall. Classical decision-tree-based models like CART and ID3 consistently lag behind, often showing the lowest accuracy—especially CART, which performs the worst in most datasets. DS08 again emerges as an outlier, where traditional models like LR, Random Forest, and XGBoost outperform NCTD and RNN significantly, suggesting that the complexity of its 26-class structure may favor simpler, more interpretable models.

Overall, this expanded comparison solidifies NCTD’s position as the top-performing and most consistent method, while also highlighting that certain datasets, particularly DS08 and DS09, may favor different approaches depending on their underlying complexity and class distribution.

## Discussion

The comparative analysis of the two experimental result tables provides insightful perspectives on the performance dynamics between NCTD, conventional machine learning methods, deep learning models, and other state-of-the-art approaches. Across both tables, NCTD consistently emerges as the top-performing method, demonstrating not only high accuracy but also remarkable stability across diverse datasets.

In the first result table, NCTD surpasses all competing methods—including IGTD, DeepInsight, REFINED, TINTO, HACNet, and Fotomics—achieving the highest accuracy in 8 out of 10 datasets and the highest average performance overall. This dominance suggests that NCTD is well-generalized and highly adaptable to varying data characteristics. While methods like HACNet and REFINED occasionally approach NCTD’s performance, they do so inconsistently. HACNet, for instance, excels in DS08 but underperforms in several other datasets, while Fotomics shows extreme variance, ranging from high performance on DS01 (96.42%) to very low performance on DS07 and DS08, indicating sensitivity to data structure.

The second result table provides a broader comparison with traditional and deep learning models. Again, NCTD demonstrates leading performance in 7 out of 10 datasets, outmatching models such as Logistic Regression, Random Forest, ID3, CART, XGBoost, and RNN. Notably, XGBoost and Random Forest are the closest contenders, often delivering competitive results but lacking consistency across all datasets. RNN, while powerful in specific instances like DS04 and DS07, suffers from severe performance degradation in DS08 (3.78%) and DS10, highlighting its vulnerability to dataset-specific characteristics, particularly those with high class diversity or unbalanced distributions.

DS08 deserves special attention, as it consistently demonstrates unusual performance patterns across both tables. The dataset’s complexity, particularly its 26-class classification structure, appears to challenge certain methods—particularly deep learning models like RNN and even NCTD—while favoring more traditional tree-based models such as Random Forest and XGBoost. This anomaly underscores the importance of dataset-specific suitability when selecting models and suggests that NCTD, while generally superior, may require adaptation or augmentation for complex multi-class problems.

In contrast, datasets like DS09 and DS10 show no clear top-performing method across the board, as accuracy remains modest and relatively close among several models. This indicates that, in cases of ambiguous or noisy data, even advanced models like NCTD may face challenges, and hybrid or ensemble strategies could offer more robust solutions.

Despite its strong performance, NCTD is not without limitations. One notable drawback is its tendency to generate large image representations, which can increase computational demands for both storage and processing. This limitation may restrict its scalability in resource-constrained environments or applications requiring real-time inference. As such, future improvements to NCTD should explore image compression strategies or lightweight alternatives to maintain performance without compromising efficiency.

Overall, the discussion reveals that NCTD stands out as a highly effective and consistent approach across a wide variety of datasets, outperforming both modern and traditional counterparts. However, it also highlights that no single method is universally optimal, and performance is strongly influenced by dataset complexity, class distribution, and feature representation. Future work should explore adaptive strategies that combine the strengths of NCTD with other models or incorporate dataset-aware mechanisms to enhance robustness across edge cases like DS08, while also addressing its computational limitations.

## Conclusion

This research introduces the Novel Algorithm for Convolving Tabular Data (NCTD), a novel technique that utilizes mathematical transformations to convert tabular data into synthetic images suitable for CNN analysis. By creating simulated spatial relationships within tabular data, NCTD enables CNNs to leverage their robust pattern recognition and feature extraction capabilities on data structures traditionally not suited for such analyses. The comprehensive evaluation demonstrates that NCTD significantly outperforms traditional machine learning algorithms and existing tabular-to-image transformation approaches across a variety of benchmark datasets. This advantage is particularly evident in complex and high-dimensional tasks, such as medical diagnostics. The experimental findings further highlight NCTD’s efficiency and versatility, showing that CNNs converge more swiftly when processing images in a 2 *N* × 2 N format. Additionally, the robustness of traditional algorithms, such as XGBoost and RF, remains notable with certain datasets, particularly those where computational efficiency and model interpretability are paramount. Future research will focus on refining NCTD’s image generation techniques and exploring hybrid models that combine the strengths of CNNs with those of traditional and other advanced machine learning approaches. Further investigation into advanced neural network architectures, as well as the integration of transfer learning, unsupervised learning, and reinforcement learning, represents promising research avenues to enhance NCTD’s adaptability and effectiveness, ensuring it remains a competitive algorithm for CNN-based classification and prediction tasks across a wide variety of datasets and tasks.Another promising research direction involves evaluating NCTD on regression tasks to broaden its applicability beyond classification.

## Electronic supplementary material

Below is the link to the electronic supplementary material.

Supplementary Material 1

## Data Availability

All datasets analyzed during this study are publicly available and have been cited accordingly in the reference section of this paper.The proprietary code used in this study can be requested from the corresponding author. The algorithm is described in detail in the methods section of this article.
