# Supplementary material for: Transforming tabular data into images via enhanced spatial relationships for CNN processing
Source: Sci Rep. 2025 May 16;15:17004. doi: 10.1038/s41598-025-01568-0 (PMC12084384; doi:10.1038/s41598-025-01568-0)
Supplement: Supplementary file 1 — Supplementary Material 1 [file 41598_2025_1568_MOESM1_ESM.docx]

**Appendices**

**Appendix *A***

| **Dataset** | **Model** | **Accuracy** | **Precision** | **Recall** | **F1-Score** |
| --- | --- | --- | --- | --- | --- |
| **DS01** | **IGTD** | 95.71% | 96% | 96% | 96% |
|  | **DeepInsight** | 94.28% | 95% | 94% | 94% |
|  | **REFINED** | 95.00% | 97% | 96% | 96% |
|  | **TINTO** | 90.71% | 91% | 91% | 91% |
|  | **HACNet** | 96.42% | 96% | 96% | 96% |
|  | **Fotomics** | 96.42% | 96% | 96% | 96% |
|  | **NCTD** | **99.28%** | **99%** | **99%** | **99%** |
| **DS02** | **IGTD** | 75.48% | 73% | 75% | 73% |
|  | **DeepInsight** | 62.25% | 58% | 62% | 57% |
|  | **REFINED** | 74.91% | 73% | 75% | 73% |
|  | **TINTO** | 74.01% | 73% | 74% | 73% |
|  | **HACNet** | 75.36% | 74% | 75% | 73% |
|  | **Fotomics** | 74.01% | 70% | 74% | 68% |
|  | **NCTD** | **78.64%** | **78%** | **79%** | **78%** |
| **DS03** | **IGTD** | 84.13% | 83% | 84% | 84% |
|  | **DeepInsight** | 84.66% | 84% | 85% | 84% |
|  | **REFINED** | 85.82% | 85% | **86%** | 85% |
|  | **TINTO** | 83.50% | 83% | 84% | 83% |
|  | **HACNet** | 85.50% | 85% | **86%** | 85% |
|  | **Fotomics** | 85.18% | 85% | 85% | 85% |
|  | **NCTD** | **86.18%** | **86%** | **86%** | **86%** |
| **DS04** | **IGTD** | 83.15% | 83% | 83% | 83% |
|  | **DeepInsight** | 84.78% | 86% | 85% | 85% |
|  | **REFINED** | 85.86% | 86% | 86% | 86% |
|  | **TINTO** | 83.69% | 84% | 84% | 84% |
|  | **HACNet** | 89.13% | 89% | 89% | 89% |
|  | **Fotomics** | 56.52% | 71% | 57% | 51% |
|  | **NCTD** | **90.22%** | **90%** | **90%** | **90%** |
| **DS05** | **IGTD** | 63.21% | 62% | 63% | 59% |
|  | **DeepInsight** | 64.31% | 47% | 64% | 52% |
|  | **REFINED** | 66.48% | 64% | 66% | 62% |
|  | **TINTO** | 65.55% | 65% | 66% | 62% |
|  | **HACNet** | 70.24% | 69% | 70% | 68% |
|  | **Fotomics** | 70.19% | 70% | 70% | 69% |
|  | **NCTD** | **71.77%** | **72%** | **72%** | **70%** |
| **DS06** | **IGTD** | 48.52% | 50% | 49% | 47% |
|  | **DeepInsight** | 43.58% | 45% | 44% | 43% |
|  | **REFINED** | 61.25% | 61% | 62% | 61% |
|  | **TINTO** | 58.57% | 58% | 59% | 57% |
|  | **HACNet** | 61.89% | **62%** | 62% | 61% |
|  | **Fotomics** | 57.35% | 57% | 57% | 57% |
|  | **NCTD** | **62.71%** | **62%** | **63%** | **62%** |
| **DS07** | **IGTD** | 95.60% | 95% | 95% | 95% |
|  | **DeepInsight** | 68.91% | 69% | 69% | 69% |
|  | **REFINED** | 91.28% | 91% | 91% | 91% |
|  | **TINTO** | 94.66% | 95% | 95% | 95% |
|  | **HACNet** | 97.09% | 97% | 97% | 97% |
|  | **Fotomics** | 49.52% | 25% | 50% | 33% |
|  | **NCTD** | **97.70%** | **98%** | **98%** | 98% |
| **DS08** | **IGTD** | 42.69% | 48% | 43% | 43% |
|  | **DeepInsight** | 87.43% | 89% | 87% | 88% |
|  | **REFINED** | 72.37% | 73% | 72% | 72% |
|  | **TINTO** | 19.93% | 19% | 20% | 17% |
|  | **HACNet** | **93.26%** | **93%** | **93%** | **93%** |
|  | **Fotomics** | 50.38% | 58% | 50% | 49% |
|  | **NCTD** | 70.64% | 86% | 85% | 85% |
| **DS09** | **IGTD** | 54.61% | 55% | 55% | 55% |
|  | **DeepInsight** | **59.42%** | **60%** | **59%** | **59%** |
|  | **REFINED** | 51.73% | 52% | 52% | 52% |
|  | **TINTO** | 58.07% | 58% | 58% | 58% |
|  | **HACNet** | 57.69% | 58% | 58% | 58% |
|  | **Fotomics** | 51.73% | 52% | 52% | 52% |
|  | **NCTD** | 55.57% | 56% | 56% | 56% |
| **DS10** | **IGTD** | 61.53% | 65% | 62% | 61% |
|  | **DeepInsight** | 60.83% | 63% | 61% | 57% |
|  | **REFINED** | 66.08% | 67% | 66% | 66% |
|  | **TINTO** | 60.48% | 60% | 60% | 60% |
|  | **HACNet** | 77.27% | 77% | 77% | 77% |
|  | **Fotomics** | 54.54% | 30% | 55% | 39% |
|  | **NCTD** | **79.37%** | **82%** | **81%** | **82%** |

**Appendix A**. Performance comparison of tabular-to-image transformation methods across various datasets.

**Appendix *B***

| **Dataset** | **Model** | **Accuracy** | **Precision** | **Recall** | **F1-Score** |
| --- | --- | --- | --- | --- | --- |
| **DS01** | **LR** | 96.42% | 96% | 96% | 96% |
|  | **CART** | 95.00% | 95% | 95% | 95% |
|  | **RF** | 95.00% | 95% | 95% | 95% |
|  | **ID3** | 92.14% | 92% | 92% | 92% |
|  | **XGBoost** | 95.00% | 95% | 95% | 95% |
|  | **RNN** | 98.57% | **99%** | **99%** | **99%** |
|  | **NCTD** | **99.28%** | **99%** | **99%** | **99%** |
| **DS02** | **LR** | 74.80% | 73% | 75% | 73% |
|  | **CART** | 67.68% | 69% | 68% | 68% |
|  | **RF** | 76.27% | 75% | 76% | 74% |
|  | **ID3** | 71.18% | 71% | 71% | 71% |
|  | **XGBoost** | 76.94% | 77% | 77% | 76% |
|  | **RNN** | 74.57% | 74% | 75% | 69% |
|  | **NCTD** | **78.64%** | **78%** | **79%** | **78%** |
| **DS03** | **LR** | 82.38% | 81% | 82% | 81% |
|  | **CART** | 80.99% | 81% | 81% | 81% |
|  | **RF** | 85.67% | 85% | 86% | 85% |
|  | **ID3** | 85.42% | 85% | 85% | 85% |
|  | **XGBoost** | 86.03% | **86%** | **87%** | **86%** |
|  | **RNN** | 84.00% | 83% | 84% | 83% |
|  | **NCTD** | **86.18%** | **86%** | 86% | **86%** |
| **DS04** | **LR** | 83.69% | 84% | 84% | 84% |
|  | **CART** | 74.45% | 75% | 74% | 75% |
|  | **RF** | 84.78% | 85% | 85% | 85% |
|  | **ID3** | 79.34% | 80% | 79% | 79% |
|  | **XGBoost** | 84.78% | 85% | 85% | 85% |
|  | **RNN** | 87.41% | 87% | 86% | 86% |
|  | **NCTD** | **90.22%** | **90%** | **90%** | **90%** |
| **DS05** | **LR** | 67.79% | 68% | 68% | 66% |
|  | **CART** | 59.56% | 60% | 60% | 60% |
|  | **RF** | 69.70% | 68% | 70% | 68% |
|  | **ID3** | 66.37% | 66% | 66% | 66% |
|  | **XGBoost** | 69.75% | 69% | 70% | 68% |
|  | **RNN** | 68.22% | 66% | 68% | 64% |
|  | **NCTD** | **71.77%** | **71%** | **71%** | **70%** |
| **DS06** | **LR** | 59.70% | 59% | 60% | 59% |
|  | **CART** | 50.18% | 50% | 50% | 50% |
|  | **RF** | 59.62% | 59% | 60% | 59% |
|  | **ID3** | 60.96% | 61% | 61% | 60% |
|  | **XGBoost** | 61.95% | **62%** | **62%** | **62%** |
|  | **RNN** | 59.88% | 59% | 60% | 59% |
|  | **NCTD** | **62.71%** | **62%** | **62%** | **62%** |
| **DS07** | **LR** | 75.20% | 75% | 75% | 75% |
|  | **CART** | 86.01% | 86% | 86% | 86% |
|  | **RF** | 95.00% | 95% | 95% | 95% |
|  | **ID3** | 85.06% | 86% | 86% | 86% |
|  | **XGBoost** | 94.72% | 95% | 95% | 95% |
|  | **RNN** | 96.28% | 96% | 96% | 96% |
|  | **NCTD** | **97.70%** | **98%** | **98%** | **98%** |
| **DS08** | **LR** | **96.41%** | **96%** | **96%** | **96%** |
|  | **CART** | 80.76% | 81% | 81% | 81% |
|  | **RF** | 94.17% | 94% | 94% | 94% |
|  | **ID3** | 81.85% | 82% | 82% | 82% |
|  | **XGBoost** | 94.87% | 95% | 95% | 95% |
|  | **RNN** | 3.78% | 0% | 4% | 0% |
|  | **NCTD** | 70.64% | 86% | 85% | 85% |
| **DS09** | **LR** | 60.19% | 60% | 60% | 60% |
|  | **CART** | 77.69% | 78% | 78% | 78% |
|  | **RF** | 69.62% | 70% | 70% | 69% |
|  | **ID3** | 75.00% | 75% | 75% | 75% |
|  | **XGBoost** | **79.80%** | **80%** | **80%** | **80%** |
|  | **RNN** | 52.30% | 57% | 52% | 44% |
|  | **NCTD** | 55.57% | 56% | 56% | 56% |
| **DS10** | **LR** | **90.55%** | **91%** | **91%** | **91%** |
|  | **CART** | 85.66% | 86% | 86% | 86% |
|  | **RF** | 87.41% | 87% | 87% | 87% |
|  | **ID3** | 81.81% | 83% | 83% | 83% |
|  | **XGBoost** | 88.11% | 88% | 88% | 88% |
|  | **RNN** | 53.49% | 75% | 53% | 38% |
|  | **NCTD** | 79.37% | 82% | 81% | 82% |

**Appendix B**. Performance comparison of conventional machine learning models across multiple datasets
